# Scaling up Studies on PEMFC Using a Modified Serpentine Flow Field Incorporating Porous Sponge Inserts to Observe Water Molecules

**DOI:** 10.3390/molecules26020286

**Published:** 2021-01-08

**Authors:** Muthukumar Marappan, Rengarajan Narayanan, Karthikeyan Manoharan, Magesh Kannan Vijayakrishnan, Karthikeyan Palaniswamy, Smagul Karazhanov, Senthilarasu Sundaram

**Affiliations:** 1 Fuel Cell Research Lab & Department of Mechanical Engineering, Nandha Engineering College, Erode 638052, India; muthupsgtech@gmail.com; 2 Fuel Cell Research Lab & Department of Electrical and Electronics Engineering, Nandha Engineering College, Erode 638052, India; rengarajan2412@gmail.com; 3 Fuel Cell Energy System Laboratory, Department of Automobile Engineering, PSG College of Technology, Coimbatore 641004, India; mkk.auto@psgtech.ac.in (K.M.); vmk.auto@psgtech.ac.in (M.K.V.); 4 Department for Solar Energy, Institute for Energy Technology (IFE), 2027 Kjeller, Norway; 5 Environment and Sustainability Institute (ESI), University of Exeter, Penryn TR10 9EZ, UK; S.Sundaram@exeter.ac.uk

**Keywords:** proton exchange membrane fuel cells (PEMFC), scaling up, porous sponge, MSSFF, EIS, water management

## Abstract

Flooding of the cathode flow channel is a major hindrance in achieving maximum performance from Proton Exchange Membrane Fuel Cells (PEMFC) during the scaling up process. Water accumulated between the interface region of Gas Diffusion Layer (GDL) and rib of the cathode flow field can be removed by the use of Porous Sponge Inserts (PSI) on the ribs. In the present work, the experimental investigations are carried out on PEMFC for the various reaction areas, namely 25, 50 and 100 cm^2^. Stoichiometry value of 2 is maintained for all experiments to avoid variations in power density obtained due to differences in fuel utilization. The experiments include two flow fields, namely Serpentine Flow Field (SFF) and Modified Serpentine with Staggered provisions of 4 mm PSI (4 mm × 2 mm × 2 mm) Flow Field (MSSFF). The peak power densities obtained on MSSFF are 0.420 W/cm^2^, 0.298 W/cm^2^ and 0.232 W/cm^2^ compared to SFF which yields 0.242 W/cm^2^, 0.213 W/cm^2^ and 0.171 W/cm^2^ for reaction areas of 25, 50 and 100 cm^2^ respectively. Further, the reliability of experimental results is verified for SFF and MSSFF on 25 cm^2^ PEMFC by using Electrochemical Impedance Spectroscopy (EIS). The use of 4 mm PSI is found to improve the performance of PEMFC through the better water management.

## 1. Introduction

Effective management of water produced as a product of Oxygen Reduction Reaction (ORR) on the cathode side is important to obtain maximum performance from PEMFC [1]. The cell performance is reduced due to the water flooding which effectively blocks the passage of protons through the membrane [2]. Increase in flooding of flow fields causes reduced performance in PEMFC at higher current densities. However, the low amount of water present causes the dehydration of the membrane which reduces the performance of the PEMFC [3]. Flow fields that produce uniform distribution of reactants and lower pressure drop due to shorter flow length yield better performance compared to serpentine flow channel [4,5,6]. The SFF produces non-uniform flow due to the presence of bends in the flow channel. The presence of bends causes a lower velocity near the bends compared to the central region of flow where the velocity is higher. Similarly, the length of the serpentine flow channel causes a higher pressure drop. Even though this helps in under rib convection, the water removal at the bottom flow channels is lower due to the reduced pressure. This in turn causes the flooding in lower flow channels and poor performance of the serpentine flow channel. Along with the operating parameters, the effective design of flow field, the surface properties and the geometrical properties (like size and shape) of flow channel play a critical role in the water accumulation and removal [7,8,9], which in turn influence the water management and performance of PEMFC. The SFF is majorly preferred among other flow field types due to the better water removal characteristics [10,11]. The performance of SFF can be further improved through the Porous Carbon Inserts (PCI) or the porous carbon molecules [12] and PSI [13]. The experimental investigations on the influence of changing the size and material of the porous inserts for 25 cm^2^ PEMFC showed that the PSI of higher sizes yield better performance than the PCI made of Vulcan carbon [13]. During scaling up, as the reaction area increases, the Ohmic resistance of the cell decreases. Because the Ohmic resistance is inversely proportional to the reaction area. However, the kinetic resistance of the cell increases since the water accumulation increases with an increase in the reaction area. This is because the reactant pressure is not high enough in the lower channels to remove the accumulated water. Hence, the performance of PEMFC drops as the reaction area increases. The larger reaction areas increase the pressure drop due to the increase in the length of the flow channel which leads to the high parasitic losses, the high mechanical stresses and the low efficiency of PEMFC. The uneven flow of reactants in the larger reaction areas causes the uneven reactions, flooding and hotspots. These are the major issues associated with the reliability and durability of PEMFC during scaling up [14,15,16,17]. The effective removal of water and enhanced pressure drop along the direction of flow can improve the performance of the PEMFC during scaling up. Hence, the water management is of prime importance, when it comes to scaling up of PEMFC. This can be achieved through the use of active or passive methods of water removal. In the case of active water removal, Electro-Osmotic (EO) pumps are inserted between the interfacial region of the rib and GDL surface area. These EO pumps continuously remove the water accumulated and prevent the flooding of the flow field [18]. However, these pumps use up to 15% of the power produced by the fuel cells. In the passive method of water removal, the flow field modifications are made to the cathode flow field such that the water formed at the cathode side is removed passively. One such arrangement is the use of porous flow field.

The use of porous flow fields made of resin-bonded carbon yielded better performance [19]. The power density gained by the use of such porous flow fields in PEMFC is higher than the conventional flow fields. However, the performance of such PEMFCs is hindered at the higher ohmic and concentration regions, due to the crossover of the reactant among the flow channels through the porous cells of flow fields. The machining of porous flow fields has proved both tedious and expensive while assembling. The use of PEMFC with such porous flow fields is a challenging task due to their brittle nature. Hence, the SFF made of non-porous graphite is modified to incorporate porous inserts made of Vulcan carbon in uniform and stagger arrangements. This modified SFF is used in order to eliminate the ill effects of reactant cross-over while retaining better water management capabilities [12]. Scaling up studies [14] with these modified flow fields is yielded better performance compared to conventional SFF of the same reaction areas. The usage of inserts is better than using the active methods such as EO pumps since the porous inserts do not require any power to operate. The use of inserts is better than other passive methods such as using porous flow fields due to the lower reactant cross-over among the channels. 

**Figure 1:**
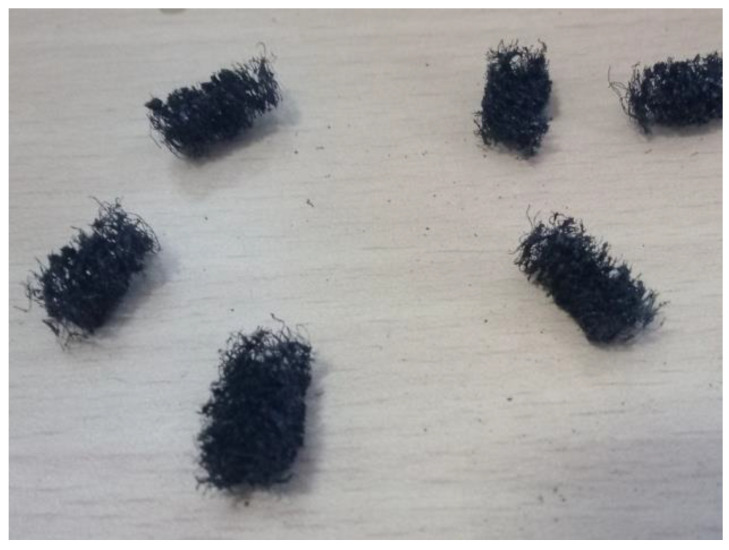
Porous sponge inserts.

The dimensions of porous inserts are specified by length × width × height. Enlarging the dimension of porous inserts from 2 mm × 2 mm × 2 mm (2 mm PSI) to 4 mm × 2 mm × 2 mm (4 mm PSI) has yielded a better performance in both PCI and PSI based on the validation with EIS. It is found that the PEMFC with PSI has yielded higher power density than the same incorporating PCI. This is mainly due to the better water absorption characteristics of PSI compared to PCI. Further, it is found that the durability of the PSI is higher in contrast to PCI which starts to dissolve in the generated water [13]. The incorporation of porous inserts increases Ohmic resistance of PEMFC due to the lower electrical conductivity of porous inserts which occupies the substantial space over the rib of the flow field. However, the overall resistance of PEMFC with 4 mm PSI is lower due to the lower charge transfer resistance as a result of better water management. Enlarging the length of PSI beyond 4 mm is not recommended due to the swelling and the deformation of the inserts. Hence, the 4 mm PSIs shown in Figure 1. are used in the present studies. 

**Figure 2:**
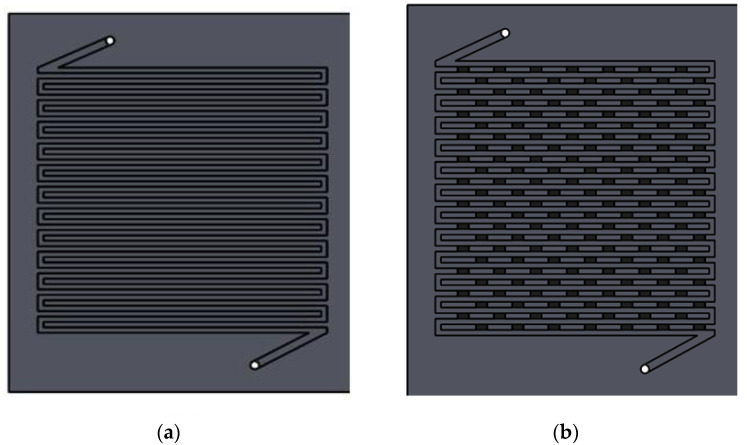
(**a**) SFF (**b**) MSSFF with reaction area of 100 cm^2^.

**Figure 3:**
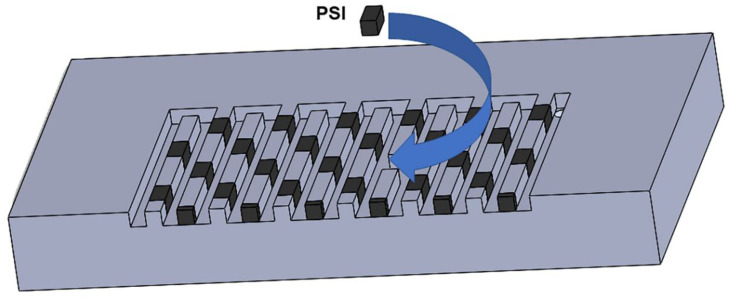
3D sectioned view of MSSFF with 4 mm PSI.

In the present study, two flow field types, namely SFF and MSSFF incorporating a Rib to Channel width ratio (R × C) of 2 × 2 are studied with various reaction areas, namely 25 cm^2^, 50 cm^2^ and 100 cm^2^. Also, the studies are carried out for various stoichiometry ranges on anode and cathode to determine the 100 % fuel/gas utilization efficiency for 25 cm^2^ PEMFC. Similarly, the stoichiometry studies are conducted for various flow fields with different reaction areas. This 2 × 2 ratio is preferred in the current study, even though it yields a slightly lesser power density compared to the 1 × 1 ratio [20,21] due to the ease machinability of slots along the rib and easy placement of porous inserts in the slots. Flow field which is capable of better water removal is ideal for the cathode [22,23,24,25]. The pressure drop in the anode is less significant compared to pressure drop in the cathode with respect to PEMFC performance. This is because the anode is not prone to flooding and the water need not be actively removed. Also, it is found that the excessive removal of water at the anode may cause the dehydration of the membrane since the water is not formed at the anode. The sources of water at the anode are only due to the humidification and diffusion from cathode to anode. However, the cathode is prone to flooding due to the formation of water which makes it ideal to employ water removal techniques in the cathode side in order to prevent excessive water build-up and flooding. Hence, the flow channel modifications are carried out only in the cathode and SFF is used in the anode side for all experiments. The depth of the flow channel is kept at 2 mm for the same reasons. In Figure 2, the designs of SFF and MSSFF are given for the 100 cm^2^ reaction area. Figure 3 shows the 3D sectioned view of the MSSFF. The machining of slots in SFF changes it to a zigzag pin flow field, whereas the insertion PSI in the slot changes it into MSSFF, thus combining the advantages of both conventional SFF and porous flow field (better water capabilities). 

## 2. Experimental Setup

### 2.1. Design and Manufacturing

The PSIs are small cuboidal bodies (size 4 mm × 2 mm × 2 mm) made from the porous carbon sponge. The porous carbon sponge is chosen due to its high porosity which enables it to absorb the water at a higher rate compared to non-porous materials. The PSI is prepared in two steps [13]. Initially, a phenolic foam is converted into a porous carbon sponge. This is done by a pyrolysis process. Commercially available phenolic foam sourced from Psychrometric Solutions (Coimbatore, India) is used for this purpose. The phenolic foam is first kept in a nitrogen atmosphere and heated steadily to 1000 °C by raising the temperature of the furnace by 100 °C each hour. This will cause the material to convert into carbon. Once the conversion is completed, the porous carbon sponge is allowed to cool to room temperature. Finally, the porous carbon sponge is sliced into a size of 4 mm × 2 mm × 2 mm to obtain the PSI.

The PSI of 90% porosity (measured using a liquid absorption method [26]) is employed in this study, as it gives enhanced performance in PEMFCs [12,14]. The permeability of porous sponge is found to be 42.62 m^2^ using the Darcy Forchheimer equation. The 4 mm PSIs are carefully positioned in slots machined on the ribs of the flow field with the aid of tweezers in a staggered arrangement so that the position of PSI is non-uniform considering adjacent ribs. A thin layer of electrically conductive glue is applied at the interface between the PSI and the graphite plate. This is to ensure that the PSI is firmly affixed to the flow plates. Otherwise, there is a possibility of hindrance to the flow of oxygen in the cathodic flow channel, if the PSI falls off from the designated position. This process of fixing the PSI in the slots machined in the ribs transforms the type of flow field from Pin type to SFF, which is vital for the better water elimination from the cathode flow field of PEMFCs.

### 2.2. Experimental Conditions

K-PAS and Bio-Logic FCT-50S fuel cell test stations are used to conduct experiments. The Bio-Logic test station (France) is used for the experiments with 25 and 50 cm^2^ PEMFCs. This Bio-Logic test station can be electronically coupled to a computer system using software named FC-Lab version 5.22. Electrical parameters such as power, voltage and current can be recorded up to 250 W, 5 V and 50 A, respectively. The maximum anode and cathode flow rates of 1 Lpm and 0.667 Lpm, respectively, can be obtained from the flow control mechanism. The K-PAS test station (India) is used for experiments on the 100 cm^2^ PEMFC, as its current measurement range is higher than that of the Bio-logic test station.

**Figure 4:**
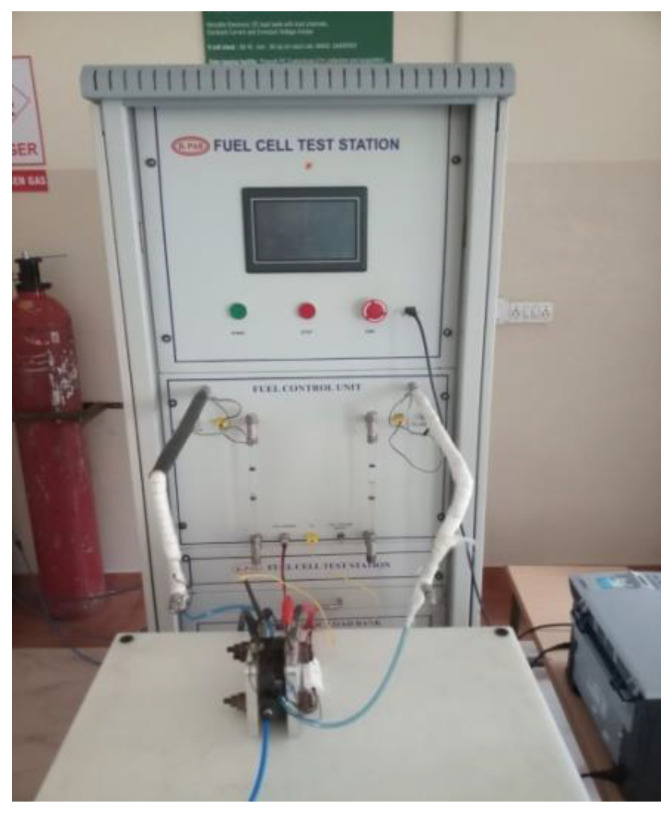
K-PAS fuel cell test station.

The K-PAS test station (shown in Figure 4) can be connected with computer using Fuel Cell software version 8.6. The membrane electrode assembly (MEA) is assembled in-house from commercially available Nafion membrane and a gas diffusion electrode (GDE) which were obtained from Fuel Cell Store (Houston, USA). The Nafion 115 membrane is used in all configurations of the present study. The GDE is made of carbon paper which is coated with Platinum and Vulcan Carbon (Mass fraction of Platinum to carbon is 40:60) as a catalyst. The loading of platinum is 0.5 mg/cm^2^.The MEA for 25 cm^2^ reaction area is prepared as follows: first, a Nafion membrane of size 8 cm × 8 cm is treated with 5% H_2_O_2_ solution for 1 h and then treated with 0.5 molar H_2_SO_4_ for 1 h at 60 °C. This is done to improve the proton conductivity of the Nafion membrane. Then, the membrane is sandwiched between two GDEs of size 5 cm × 5 cm on both sides. This is done by pressing with 50 kgf/cm^2^ of pressure over a period of 3 min at 150 °C. The same procedure is followed for the preparation of MEAs for the reaction area of 50 cm^2^ and 100 cm^2^. Copper plates are utilized as anode and cathode current collectors. Aluminum plates are utilized as endplates to hold the components together. The hydrogen supplied is of 99.99% purity and oxygen supplied is of medical grade to avoid the poisoning of the catalyst materials by carbon monoxide and sulphur.

Among the various methods namely, the current interrupt method, AC resistance method, the high-frequency resistance method [27,28,29,30] and EIS method to compare the performance based on resistance, EIS is used in this study as it can measure both real and imaginary values of impedance along with both magnitude and phase. EIS is a method that can be used to monitor and test any system that responds to current like fuel cells, electrolysers and batteries. EIS is a method of investigating the properties of an electrochemical system through impedance. The advantage of EIS is that it allows us to study both the surface and bulk properties of the system under study though analogies to circuit elements separating out the responses of different mechanisms in our system by the time constant of their response to perturbation. In EAS the response of the system to alternating potential or current signals over a range of frequencies are measured. Practically, this means that we can measure diffusion coefficient, kinetic parameters and electrolyte resistance to our systems.

The MEA is properly humidified for obtaining maximum performance by using the activated catalyst sites. The membrane activation is done by the following procedure for all reaction areas based on past experimental studies. Initially, a voltage of 0.6 V is constantly maintained for 60 min. Then, the voltage is alternated between 0.4 V and 0.7 V for each 20-min interval by using a looping technique until the current density attained reaches a maximum value. In the end, the PEMFC is subjected to the current pulse of 190–210 mA/cm^2^ till the voltage stabilizes. The scaling up study is conducted after the activation of the MEA.

The software used for interfacing the fuel cell test stations with the computer offers various options for experiments. Among the different types of experiments, the current scan method and the voltage scan method are used. The current scan method enables the user to obtain data on various parameters such as voltage, power, power density, etc. over a range of preset values of current which can be incremented or decremented in small steps. The voltage scan method on the other hand allows the user to collect data on parameters like current, current density, power, power density for pre-determined values of voltage in a given range of incremental steps.

Experimental data are acquired after reaching the steady-state condition. For every voltage, the flow rate pertaining to 2.0 stoichiometry is given, and the fuel cell is permitted to function until there is no variation in current for 60 min indicating that steady-state of cathode has been attained. This is because the water formation, oxygen consumption, current density and water removal are closely related to each other. Better reactant consumption results in a higher amount of water generation which leads to lower current density due to increased kinetic resistance which will tend to reduce the reactant consumption. At the same time, better water removal leads to higher current density which in turn produces more water due to higher reactant consumption. This process is cyclically repeated until the water generation and removal are balanced which produce constant current density.

## 3. Results and Discussions

The performance of PEMFC is experimentally investigated for two flow fields (SFF and MSSFF) on the cathode side with three different reaction areas, namely 25, 50 and 100 cm^2^. The SFF is used on anode side in each configuration in this study. The rib to channel width ratio (R × C) is maintained at 2 × 2 and the height of the flow channel is maintained at 2 mm for all the flow field configurations. Before carrying out the experimentation on the effect of different reaction areas of flow field, the stoichiometry study has been carried out for various stoichiometry ranges on anode and cathode to ensure maximum reactant utilization efficiency for 25 cm^2^ PEMFC.

### 3.1. Stoichiometry Study

**Figure 5:**
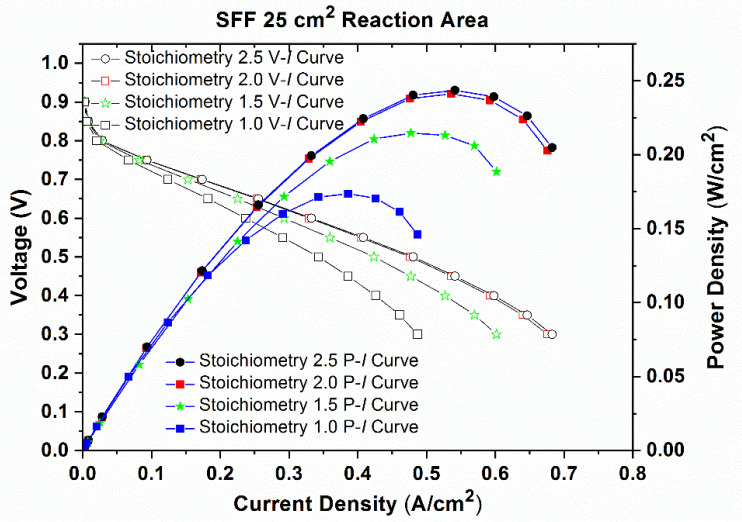
Performance (P–I) curves and Polarization (V–I) curves for SFF with 25 cm^2^ reaction area for various stoichiometry values.

Initially, the maximum current density is found by allowing the reactants to flow at maximum flow rates on both anode and cathode for every flow channel configuration. Then, the stoichiometry is found from the maximum current density using the Nernst equation. Now, the performance of PEMFC is measured for different stoichiometry values starting from 1.0 to 2.5 in increments of 0.5. The performance and polarization curves for the same is shown in Figure 5.

From Figure 5, it can be observed that the power density and current density attained are increased with increase in stoichiometry. However, the rate of increase in power density and current density is low at higher stoichiometries. The maximum power densities and current densities achieved at each stoichiometry are given in Table 1.

From Table 1, it is clear that the increase in maximum power density is negligible for increase in stoichiometry value from 2.0 to 2.5 compared to previous cases. The similar experiments are conducted for different flow fields and it is found that the stoichiometry of 2.0 is suitable to conduct performance studies. Hence, the scaling up studies are conducted with the stoichiometry value of 2.0.

The temperature of the cell, reactants and humidifier is maintained at 313 K for the PEMFC with 25 cm^2^ reaction area. The experimentation is carried out for various stoichiometry values on the anode and cathode to determine the 100% fuel/gas utilization efficiency. Based on the stoichiometry studies conducted for SFF with 25 cm^2^, the stoichiometry value of 2 is selected for both anode and cathode for all reaction areas. The operating pressure is maintained at 1 bar. The experiments for scaling up studies are conducted under same experimental conditions to compare the performance of PEMFC while increasing the reaction areas. These experiments are conducted in order to study the outcome of modifying the flow field type from SFF to MSSFF, while scaling up the reaction area of PEMFC from 25 cm^2^ to 50 cm^2^ and then to 100 cm^2^. The same experimental conditions are used in EIS studies to avoid ambiguity. The experiments have been repeated for three times to ensure repeatability and the final values are represented here.

### 3.2. Effect of Flow Rate, Pressure Drop and Backpressure

In order to assess the effect of flow rate on the performance of SFF, the flow rate equal to that of the MSSFF is given. The flow rate of the SFF at 3.0 stoichiometry is almost equal to the flow rate of the MSSFF at 2.0 stoichiometry. The peak power densities of SFF at stoichiometries of 2.0, 2.5 and 3.0 are 0.242 W/cm^2^, 0.244 W/cm^2^ and 0.246 W/cm^2^ respectively, whereas the peak power density of MSSFF is 0.424 W/cm^2^ at stoichiometry of 2.0. The results are imperative, the change in performance due to fuel flow rate between serpentine and the modified serpentine flow field is small. Hence, the flow rate alone could not be demarcated for the enhancement in performance of MSSFF.

In SFF, the pressure drop is high, in the order of 1675 Pa. The MSSFF has a lower pressure drop due to enhanced reactant crossover, in the order of 778 Pa as measured by a pressure transducer. Thus, the pressure drop in MSSFF is lesser than that of SFF which in turn results in better performance [6,17]. This is in concurrence with the results of the numerical studies conducted.

One of the important parameters affecting PEMFC performance is the backpressure. Experimental studies indicate that the performance of PEMFC considerably increases with increase in backpressure. The increase in performance due to the increase in backpressure may be attributed to the reactants forced against catalyst sites and increase the residence period of reactants in the flow channel.

In order to study the effect of backpressure in both SFF and MSSFF, a comparison study is carried out in both flow fields with and without backpressure. A backpressure of 1.5 bar is given at both the anode and cathode side in order to maintain the same condition on either side of the MEA. The results of the same are given in Table 2.

From Table 2, it is observed that the power density in SFF with backpressure is 0.263 W/cm^2^ whereas the same obtained from SFF without backpressure is 0.242 W/cm^2^. The power densities obtained by MSSFF with and without backpressure are 0.474 W/cm^2^ and 0.42 W/cm^2^ respectively. Introducing backpressure in SFF increased the performance by 8.67% compared to SFF without backpressure. Similarly, MSSFF showed a 12.85% increase in power density when backpressure is given.

Even though an increase in power density is observed while increasing the backpressure, it is found that the induction of backpressure reduces the water removal capacity of the flow field since PEMFC operated with backpressure provides a higher resistance to the flow of reactants compared to PEMFC operated without backpressure. This causes accumulation of water in the flow field leading to frequent flooding. This demands frequent purging to get rid of surplus water in the flow field in order to maintain the performance of PEMFC. Hence, the backpressure is not considered in the scaling up studies in order to avoid flooding.

### 3.3. Effect of Flow Field Modification for Various Reaction Areas of PEMFC

**Figure 6:**
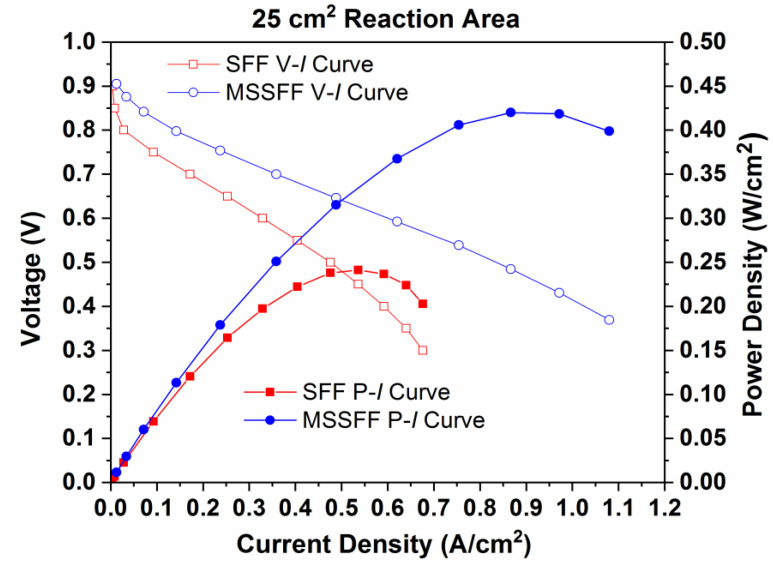
Performance (P–I) curves and Polarization (V–I) curves for SFF and MSSFF on cathode side with 25 cm^2^ reaction area.

Figure 6 exhibits the performance (P–I) curves and polarization (V–I) curves for the 25 cm^2^ PEMFC employing SFF and MSSFF. The power densities gained for PEMFCs with SFF and MSSFF are 0.242 W/cm^2^ and 0.420 W/cm^2^, respectively, and the current densities gained are 0.676 A/cm^2^ and 1.08 A/cm^2^, respectively. The increase in power density owing to the use of MSSFF on the cathode side instead of SFF is 73.55%.

**Figure 7:**
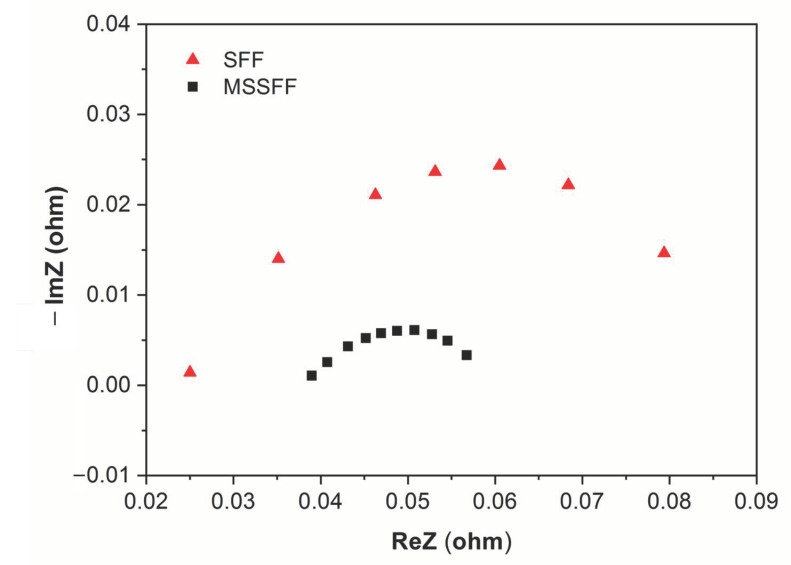
Nyquist plot of PEMFC for SFF and MSSFF.

Figure 7 exhibits the Nyquist plot of PEMFC for SFF and MSSFF derived from EIS studies. At 0.45 V, the experiment is conducted due to the occurrence of high water lodging in this particular cell potential. The frequency range between 10 kHz and 10 MHz is used for conducting the EIS experiment. The amplitude of the alternating current (AC) is maintained at 5% of the direct current (DC).

The start of the semi-circle indicates Ohmic resistance (R_O_) and the diameter of the semi-circle when completed indicates kinetic resistance (R_k_) in the Nyquist plot. It can be seen that the Ohmic resistance of the SFF is less than that of MSSFF. However, the radius of the EIS curve is lower for the MSSFF with 4 mm PSI compared to that of SFF. This implies that the kinetic resistance is very high for the SFF due to water flooding between the interface of the GDL and the rib surface of the flow field.

**Figure 8:**
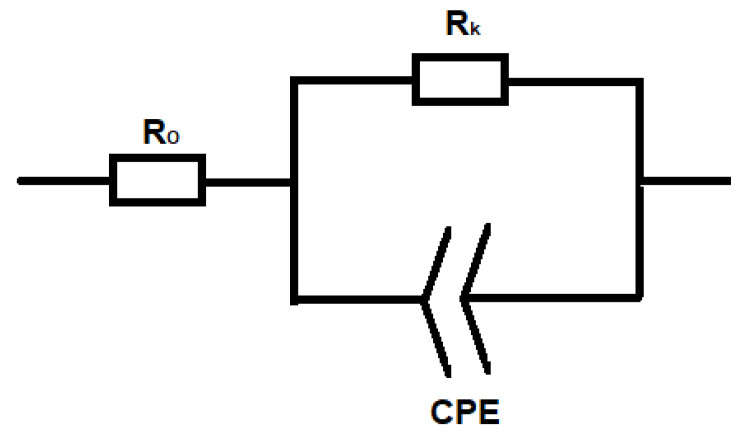
Equivalent Circuit of PEMFC for SFF and MSSFF.

The equivalent circuit obtained from the Nyquist plot is shown in Figure 8 and the values of ohmic resistance and kinetic resistance for SFF and MSSFF are given in Table 3. The value of constant phase element (CPE) is of little importance to this study, hence it is not included in Table 3. However, it is included in Figure 8 for the completeness of the equivalent circuit.

From Table 2, it is observed that Ohmic resistances of 0.025 Ω and 0.038 Ω are measured for the SFF and MSSFF, respectively. The Ohmic resistance of PSI material having air voids is more than the solid graphite material of the SFF. However, the kinetic resistance of the MSSFF is found to be 0.023 Ω which is comparatively lower to the SFF which has a kinetic resistance of 0.0625 Ω. This is because the water lodging between the interface region of the GDL and the rib surface is more effectively removed in MSSFF than that of SFF. This in turn causes the total resistance for MSSFF (0.061 Ω) to be lower than that of SFF (0.0865) resulting in higher current for same voltage since the current at a given voltage is inversely proportional to resistance according to Ohm’s law. These above reasons justify the results shown in Figure 6. From the above results, it is evident that the adoption of PSI leads to better water removal and yields the maximum power density. Hence, the same arrangement is used in the scaling up studies for PEMFCs with active areas of 50 cm^2^ and 100 cm^2^.

**Figure 9:**
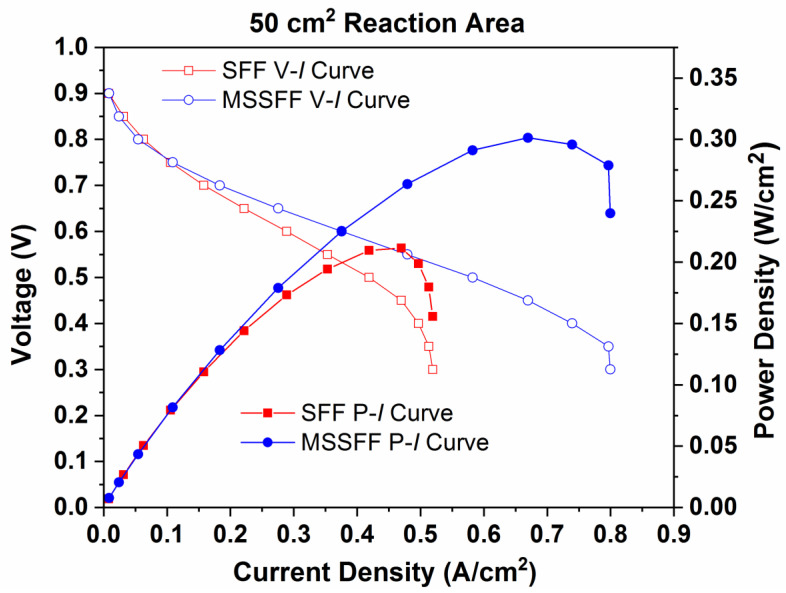
Performance (P–I) curves and Polarization (V–I) curves for SFF and MSSFF on cathode side with 50 cm^2^ reaction area.

Figure 9 exhibits the performance (P–I) curves and polarization (V–I) curves for 50 cm^2^ PEMFC employing SFF and MSSFF on the cathode side. The power densities gained for SFF and MSSFF are 0.213 W/cm^2^ and 0.298 W/cm^2^, respectively, and the current densities attained are 0.519 A/cm^2^ and 0.8 A/cm^2^, respectively. The increase in power density owing to the use of MSSFF on the cathode side instead of SFF is 39.90%.

**Figure 10:**
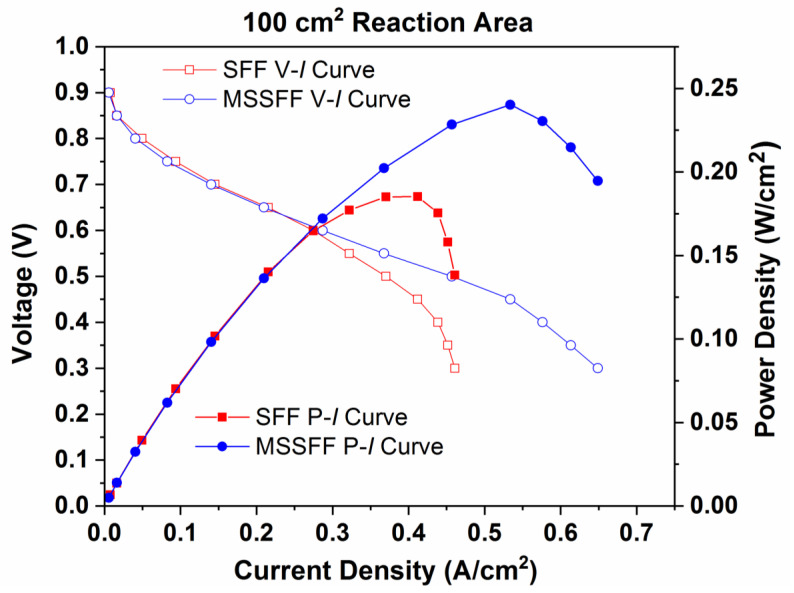
Performance (P–I) curves and Polarization (V–I) curves for SFF and MSSFF on cathode side with 100 cm^2^ reaction area.

Figure 10 exhibits the performance (P–I) curves and polarization (V–I) curves for 100 cm^2^ PEMFC employing SFF and MSSFF on the cathode side. The power densities attained for the SFF and MSSFF are 0.171 W/cm^2^ and 0.232 W/cm^2^, respectively, and the current densities attained are 0.461 A/cm^2^ and 0.65 A/cm^2^, respectively. The increase in power density owing to the use of MSSFF on the cathode side instead of SFF is 35.74%.

Modifying the flow field type from SFF to MSSFF by machining slots of size 4 mm × 2 mm × 2 mm on the ribs of the flow field and incorporating 4 mm PSI in the slots increases the performance of PEMFC irrespective of the reaction area. The machining of slots along the ribs of SFF converts it into a zigzag pin flow field. The placement of PSI on the slots of zigzag pin flow field converts it to MSSFF with porous pathways for effective water management. This enables the MSSFF to inherit the advantages of both SFF and the porous flow field. This in turn facilitates the effective removal of water accumulated in the interfacial region of the GDL and rib surface while minimizing crossover of reactants among the channels.

In addition to this, the positioning of PSI in the flow field enables them to absorb the water formed in the interfacial region of the GDL and rib surface, due to the porous nature and capillarity action of the pores present in the PSI. After the PSI is completely saturated with water, the excess water is dripping down to the adjacent flow channel due to the gravity, instead of going back to the GDL/rib interface. This water is either carried away by the flow of reactants or absorbed on the next PSI of the adjacent rib. This process is continuously repeated until the excess water is removed from the flow field. Further, the use of a staggered provision of inserts in MSSFF provides globalized removal of water in contrast to conditions in localized removal of water in the uniform provision of inserts. This allows MSSFF to achieve the higher performance compared to other flow fields through effective water management.

### 3.4. Influence of Scaling Up for Different Flow Field

**Figure 11:**
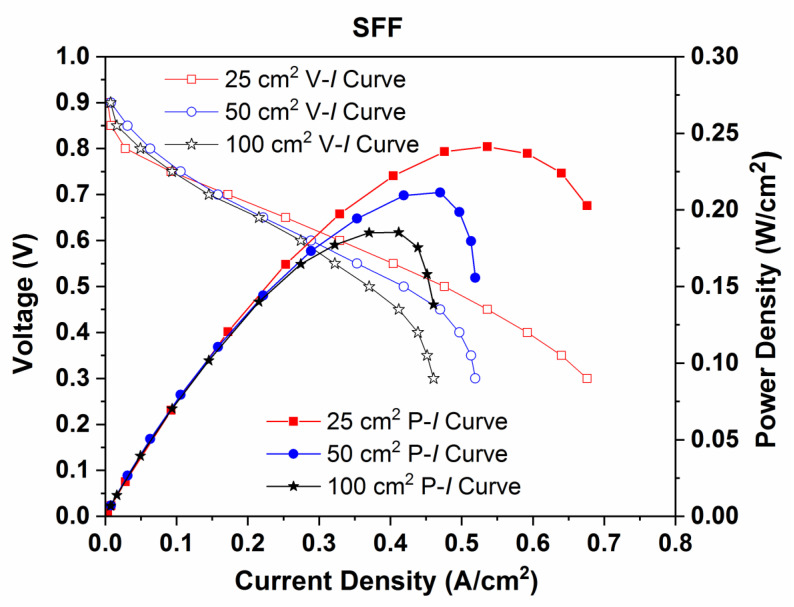
Performance (P–I) curves and Polarization (V–I) curves for PEMFC with reaction areas of 25, 50 and 100 cm^2^ employing SFF.

Figure 11 exhibits the performance (P–I) curves and polarization (V–I) curves for PEMFCs with reaction areas of 25, 50 and 100 cm^2^ employing SFF on both the anode and cathode sides. The power densities gained are 0.242 W/cm^2^, 0.213 W/cm^2^ and 0.171 W/cm^2^ for PEMFCs with reaction areas of 25, 50 and 100 cm^2^, respectively. The corresponding values of current densities gained are 0.676 A/cm^2^, 0.519 A/cm^2^ and 0.461 A/cm^2^. The power densities decrease by 11.98% and 29.33%, while doubling and quadrupling the reaction areas of PEMFC, respectively.

**Figure 12:**
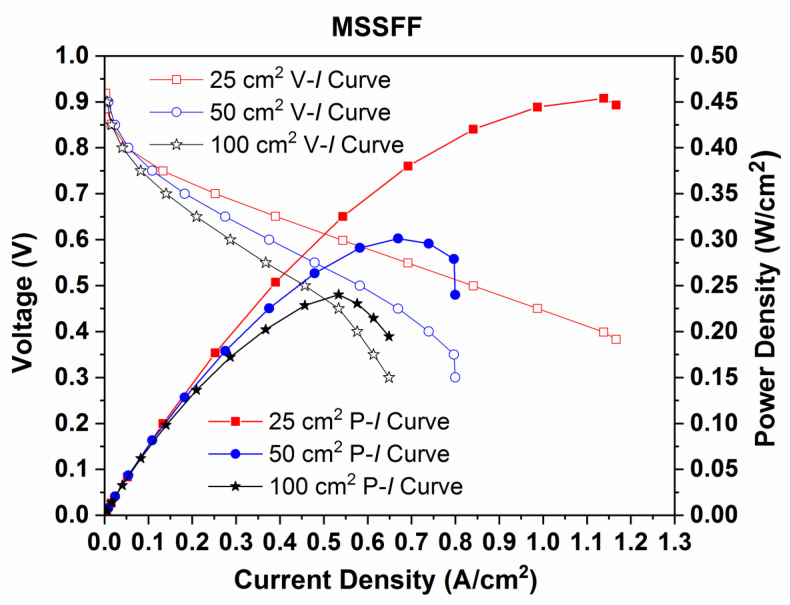
Performance (P–I) curves and Polarization (V–I) curves of PEMFCs with reaction areas of 25, 50 and 100 cm^2^ employing MSSFF.

Figure 12 exhibits the performance (P–I) curves and polarization (V–I) curves for PEMFCs with reaction areas of 25, 50 and 100 cm^2^ employing MSSFF on the cathode side. The power densities gained are 0.420 W/cm^2^, 0.298 W/cm^2^ and 0.232 W/cm^2^ for PEMFC with reaction areas of 25, 50 and 100 cm^2^, respectively. The corresponding current densities attained are 1.08 A/cm^2^, 0.8 A/cm^2^ and 0.65 A/cm^2^, respectively. The power density decreases by 29.04%, while doubling the reaction area and decreases by 44.76%, while quadrupling the reaction areas of PEMFC.

The power density attained is the highest for PEMFC with 25 cm^2^ reaction area, as both the distance traveled by the reactant and the length of the flow channel is shorter. In the case of SFF, the pressure drop is higher which in turn results in better diffusion and the forced convection in the direction normal to flow of reactants. Consequently, the time spent by reactants on the catalyst sites is more. This yields better performance. However, increasing the reaction area of PEMFC makes the process of removing the accumulated water more difficult. Thus, the performance of PEMFC with higher reaction areas begins to drop significantly. In the case of MSSFF, some quantity of the accumulated water is removed by PSI, which results in overall better performance when compared to SFF of the same size. However, the increase in performance of the modified flow field design is not linear, while scaling up the PEMFC. This may be caused by the trade-off obtained between the water removed by PSI and the reactant cross-over due to the insertion of PSI. Table 4 consolidates the effects of changing flow field designs and increasing the reaction areas of PEMFC.

**Figure 13:**
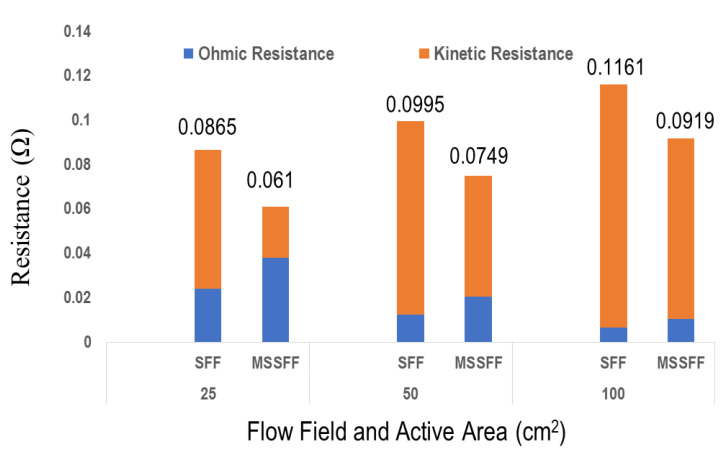
Resistances obtained from EIS studies for PEMFCs with reaction areas of 25, 50 and 100 cm^2^ employing SFF and MSSFF.

EIS studies are conducted for 50 cm^2^ and 100 cm^2^ reaction area PEMFCs similar to 25 cm^2^ reaction area PEMFC in order to validate the results shown in Table 3. The resistances obtained are plotted in Figure 13. 

From Figure 13, it is found that the Ohmic resistance increases in MSSFF compared to SFF due to addition of PSI. However, it is found that the kinetic resistance of MSSFF is lower in all active areas due to the better removal of water by the presence of PSI which in turn mitigates the water flooding. Also, it is found that the decrease in kinetic resistance in MSSFF is always more than the increase in ohmic resistance in all the reaction areas. It results in lower total resistance of MSSFF compared to the total resistance of SFF in all three reaction areas which in turn increases the current density attained from PEMFC. This is in concurrence with the data available in Table 4. This is an indication of better performance of MSSFF compared to SFF. 

It is also found that irrespective of flow field design, the total resistance increases when the reaction area is increased. This is due to the better water removal in the lower reaction areas. However, the MSSFF is found to be having the better performance in all the reaction areas due to the better water management compared to SFF. Further, the incorporation of PSI mitigates the flooding in flow channels.

## 4. Conclusions

The experimental investigations on the performance of PEMFCs are conducted by scaling the reaction areas from 25 cm^2^ to 50 cm^2^ and then 100 cm^2^ using two cathode flow fields namely SFF and MSSFF while retaining SFF on anode side.

- Adoption of 4 mm PSI with the porosity of 90% on the cathode side of PEMFC using MSSFF yields better performance than SFF on all the three reaction areas. The increments in power density of MSSFF compared to SFF are 73.55%, 39.9% and 35.74% for PEMFCs with reaction areas of 25, 50 and 100 cm^2^, respectively. Further, the reliability of the experimental results is verified for SFF and MSSFF on 25 cm^2^ PEMFC by using EIS.
- The adoption of 4 mm PSI in MSSFF on the cathode side of PEMFC has a significant effect on reducing the water flooding associated with the scaling of PEMFCs due to the enhanced water management properties.
- The power density gained is the maximum for PEMFCs employing MSSFF irrespective of the reaction area. This is due to the effective removal of water accumulated in the interfacial region of the GDL and the rib surface of the flow field. Hence, it is concluded that MSSFF on the cathode flow field is better than SFF for better management of water and the higher performance.

## Figures and Tables

**Table 1:** Increase in power density for various stoichiometry values on 25 cm^2^ PEMFC with SFF.

Stoichiometry	Maximum Current Density (A/cm^2^)	Maximum Power Density (W/cm^2^)	Percentage Increase in Power Density Compared to Previous Stoichiometry
1.0	0.487	0.174	-
1.5	0.602	0.215	23.56
2.0	0.676	0.241	12.09
2.5	0.683	0.244	1.24

**Table 2:** Effect of backpressure on power density in 25 cm^2^ PEMFC with SFF and MSSFF.

Flow Field	Maximum Power Density (W/cm^2^)		Increase in Power Density Due to Backpressure (%)
	without Backpressure	with Backpressure	
SFF	0.242	0.676	8.67
MSSFF	0.213	0.519	12.85

**Table 3:** Values of ohmic resistance and kinetic resistance for SFF and MSSFF.

Flow Field	Ohmic Resistance (Ω)	Kinetic Resistance (Ω)	Total Resistance (Ω)
SFF	0.024	0.0625	0.0865
MSSFF	0.038	0.023	0.061

**Table 4:** Influence of changing flow field designs and increasing the reaction areas of PEMFC.

Reaction Area (cm^2^)	SFF		Decrease in Power Density Due to Scaling Up of Reaction Area Compared to 25 cm^2^(%)	MSSFF		Decrease in Power Density Due to Scaling Up of Reaction Area Compared to 25 cm^2^ (%)	Increase in Power Density of Mssff Compared to Sff (%)
	Peak Power Density (W/cm^2^)	Peak Current Density (A/cm^2^)		Peak Power Density (W/cm^2^)	Peak Current Density (A/cm^2^)		
25	0.242	0.676	-	0.420	1.08	-	73.55
50	0.213	0.519	11.98	0.298	0.8	29.04	39.90
100	0.171	0.461	29.33	0.232	0.65	44.76	35.74

## Sample Availability

Its research article based on Flow fields studies on fuel cells.

## Abbreviations

The following abbreviations are used in this manuscript:

AC	Alternating Current
CL	Catalyst Layer
CPE	Constant Phase Element
DC	Direct Current
EIS	Electrochemical Impedance Spectroscopy
GDE	Gas Diffusion Electrode
GDL	Gas Diffusion Layer
MEA	Membrane Electrode Assembly
MSSFF	Modified Serpentine with Staggered provisions of 4 mm porous sponge insert Flow Field
ORR	Oxygen Reduction Reaction
PCI	Porous Carbon Inserts
PEMFC	Proton Exchange Membrane Fuel Cell
PSI	Porous Sponge Inserts
RxC	Rib to Channel width ratio
SFF	Serpentine Flow Field
